# Thyroid Dysfunction Following Tirzepatide Use in a Post-thyroidectomy Patient on Stable Levothyroxine Therapy: A Case Study

**DOI:** 10.7759/cureus.106680

**Published:** 2026-04-08

**Authors:** Emily W Adams, Alex Somers, Emmanuel Garrido-Cortes, Bobby Wrights

**Affiliations:** 1 Research, Alabama College of Osteopathic Medicine, Dothan, USA; 2 Research, Edward Via College of Osteopathic Medicine, Auburn, USA; 3 Internal Medicine, Infirmary Health Diagnostic and Medical Clinic, Mobile, USA

**Keywords:** drug-related side effects and adverse reactions, glp-1 agonist, levothyroxine overdose, post-thyroidectomy management, thyroidectomy complications, weight loss counselling

## Abstract

Tirzepatide, a dual glucagon-like peptide-1 (GLP-1)/glucose-dependent insulinotropic polypeptide (GIP) receptor agonist, is increasingly prescribed for obesity and glycemic control. Its effects on gastric emptying, appetite regulation, and rapid weight loss may influence thyroid hormone homeostasis and the pharmacokinetics of narrow-therapeutic-index medications such as levothyroxine. Emerging clinical experience suggests that GLP-1-based therapies can suppress thyroid-stimulating hormone (TSH), alter thyroid hormone dynamics, and precipitate symptoms of thyrotoxicosis in patients receiving thyroid hormone replacement. As use of incretin therapies expands, understanding their potential interaction with thyroid physiology is increasingly important.

We describe a 52-year-old woman with a history of thyroidectomy complicated by postoperative hypothyroidism, maintained on stable doses of levothyroxine for several years. After six months of tirzepatide therapy, she experienced 48 pounds of intentional weight loss and developed dizziness, tachycardia, weakness, and confusion. Laboratory evaluation showed a markedly suppressed TSH and elevated free T4, consistent with significant over-replacement with levothyroxine. She required emergent dose reduction and supportive care, with gradual resolution of symptoms after adjustment of her thyroid regimen.

Key contributing factors included substantial weight loss without proportional levothyroxine dose reduction, delayed gastric emptying altering levothyroxine absorption, and absence of endogenous thyroid reserve following thyroidectomy. These changes created a clinical environment highly susceptible to pharmacologic fluctuations and rapid shifts into iatrogenic thyrotoxicosis. Additionally, GLP-1 therapy-associated decreases in blood pressure and appetite likely amplified her acute presentation.

This case highlights the importance of vigilant monitoring in post-thyroidectomy patients who begin tirzepatide or other incretin-based medications. Clinicians should anticipate the need for levothyroxine dose adjustments as weight decreases and gastrointestinal motility changes. Early follow-up, routine thyroid function testing, and prompt evaluation of new cardiovascular or neurocognitive symptoms are essential to prevent avoidable morbidity related to drug-hormone interactions.

## Introduction

Tirzepatide, a dual glucose-dependent insulinotropic polypeptide (GIP) agonist and glucagon-like peptide-1 receptor agonist (GLP-1 RA), has rapidly emerged as a leading agent for obesity and type 2 diabetes mellitus due to its potent effects on weight reduction, appetite suppression, glycemic control, and cardiometabolic outcomes. As its use expands, increasing attention has turned toward its effects on thyroid function, particularly in individuals reliant on exogenous thyroid hormone replacement. Rapid pharmacologic weight loss can destabilize levothyroxine requirements because weight-based dosing is highly sensitive to changes in body mass, placing patients at increased risk of over-replacement [1]. Thyroid dysfunction is also common among individuals with metabolic disease, with epidemiologic data demonstrating increased rates of hypothyroidism in patients with diabetes, obesity, autoimmune disease, and prior bariatric surgery [2].

Emerging evidence suggests that GLP-1 receptor agonists, including semaglutide, liraglutide, and tirzepatide, may influence thyroid laboratory values, thyroid-stimulating hormone (TSH) secretion, and thyroid pharmacokinetics, with several mechanisms proposed [2-4]. Additional thyroidal effects, including GLP-1 receptor stimulation of thyrotropin-releasing hormone (TRH)-expressing neurons in the hypothalamic paraventricular nucleus, suggest a potential central influence on the hypothalamic-pituitary-thyroid (HPT) axis [5,6]. Case reports have described tirzepatide-associated thyrotoxicosis in patients on stable levothyroxine doses [1,5]. Real-world cohort studies have further shown that greater GLP-1-mediated weight loss is associated with greater reductions in TSH among patients receiving levothyroxine therapy [7]. A recent meta-analysis of randomized controlled trials additionally demonstrated an increased risk of thyroid disorders among GLP-1 RA users [8].

This case involves a post-thyroidectomy patient who developed severe biochemical hyperthyroidism following significant tirzepatide-associated weight loss without levothyroxine adjustment. The case underscores the need for proactive levothyroxine dose modification and close thyroid surveillance when GLP-1/GIP agonists are initiated in thyroid-dependent patients.

## Case presentation

A 52-year-old woman (height 1.626 m, weight 68.2 kg) presented to the emergency department on October 14, 2025, with complaints of dizziness, generalized weakness, progressive confusion, and concern for dehydration. Recent history was pertinent for 48-lb intentional weight loss over the preceding six months, which she attributed to ongoing treatment with the GLP-1/GIP receptor agonist tirzepatide.

Past medical and surgical history was notable for breast cancer (stage 2, hormone-receptor+), currently managed medically with letrozole and fezolinetant (Veozah) since May 2025, a gastric bypass procedure in 2022, and a total thyroidectomy in 2017 followed by chronic postoperative hypothyroidism managed with long-term levothyroxine therapy. Additional history included Graves’ disease diagnosed in 2010, bipolar disorder, and Goldenhar syndrome with dermoid tumor excision at two years of age. Her chronic medications consisted of levothyroxine 175 mcg daily (stable for more than five years), weekly tirzepatide 7.5 mg (last administered six days before presentation), letrozole, fezolinetant, and intermittent use of a bariatric multivitamin. She denied recent iodinated contrast exposure, medication nonadherence, or changes in dosing.

On arrival, vital signs demonstrated mild tachycardia and low-normal blood pressure (Table 1). Physical exam revealed a fatigued and mildly confused female with physical findings consistent with clinical dehydration - dry, cracked lips and delayed capillary refill. Additional physical findings revealed thinning hair and mild tachycardia. No tremor, exophthalmos, or palpable thyroid tissue was appreciated due to prior thyroidectomy. Neurologic evaluation was non-focal.

**Table 1:** Vital Signs on Presentation BMI: body mass index; mmHg: millimeters of mercury; bpm: beats per minute.

Parameter	Value	Reference Range
Blood pressure	104/64 mmHg	90–120 / 60–80 mmHg
Heart rate	105 bpm	60–100 bpm
Body Mass Index (BMI)	25.4 kg/m²	18.5–24.9 kg/m²

**Figure 1:**
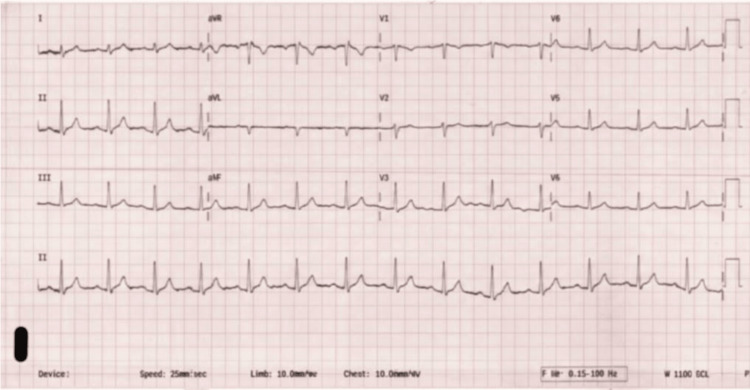
Emergency Department Electrocardiogram From Initial Presentation. Twelve-lead electrocardiogram obtained during emergency department evaluation demonstrating normal sinus rhythm with normal axis and intervals, without acute ST-segment or ischemic changes.

Initial laboratory studies demonstrated marked thyroid hormone derangement, with a suppressed TSH and elevated free thyroxine (T4), along with mild abnormalities in iron indices and vitamin B12 levels (Table 2). Complete blood count (CBC) values were notable only for a mean corpuscular hemoglobin concentration (MCHC) of 32.5%. Iron studies revealed a transferrin level of 193 mg/dL and total iron-binding capacity (TIBC) of 245 μg/dL. Vitamin B12 was elevated at 1,522 pg/mL. Other laboratory studies, including electrolytes, renal function, micronutrient levels, and urinalysis, were within normal limits. The electrocardiography, shown in Figure 1, displayed normal sinus rhythm. 

**Table 2:** Laboratory Findings at Time of Emergency Room Visit

Test	Result	Reference Range
Thyroid Studies		
Thyroid-stimulating hormone (TSH)	<0.005 μIU/mL	0.4–4.5 μIU/mL
Free Thyroxine (T4)	2.43 ng/dL	0.8–1.8 ng/dL
Mean corpuscular hemoglobin concentration	32.5%	32–36%
Transferrin	193 mg/dL	200–360 mg/dL
Total iron-binding capacity	245 μg/dL	250–450 μg/dL
Vitamin B12	1,522 pg/mL	200–900 pg/mL

The clinical picture was consistent with iatrogenic hyperthyroidism, attributed to excessive levothyroxine replacement in the context of significant tirzepatide-associated weight loss.

The patient was treated with an intravenous 0.9% sodium chloride bolus and maintenance infusion for volume repletion. Her levothyroxine dose was reduced from 175 mcg to 100 mcg daily, and daily use of a bariatric multivitamin was recommended. The care team also advised considering extended dosing intervals of tirzepatide to every other week. Outpatient follow-up with her primary care provider was arranged, including repeat thyroid function testing (TSH, free T4, and free T3), basic metabolic panel, and lipid panel.

At her primary care visit on November 10, 2025, the patient reported marked improvement in her symptoms. Repeat laboratory testing was pending at that time, and further levothyroxine dose adjustments were anticipated based on upcoming results.

## Discussion

This case illustrates how tirzepatide can significantly destabilize thyroid hormone regulation in a post-thyroidectomy patient by altering weight-based levothyroxine requirements, gastrointestinal absorption, and central HPT-axis function.

Rapid tirzepatide-induced weight loss reduced levothyroxine requirements

Levothyroxine dosing in athyreotic patients is approximately 1.6 μg/kg/day, making it highly sensitive to changes in body weight [7]. Our patient experienced a 48-lb (21.8-kg) weight loss over six months of tirzepatide therapy without any adjustment in her levothyroxine dose. Similar cases of tirzepatide-induced thyrotoxicosis have been reported, including a 2024 report documenting severe hyperthyroidism in a patient on stable levothyroxine following rapid tirzepatide-associated weight loss [1]. Real-world cohort data further demonstrate that greater GLP-1-mediated weight loss is associated with greater TSH suppression in levothyroxine-treated patients [7].

In this case, progressive weight loss transformed a previously appropriate levothyroxine dose into significant over-replacement, producing marked hyperthyroidism.

Delayed gastric emptying altered levothyroxine absorption

Tirzepatide slows gastric emptying, a well-characterized class effect of GLP-1 receptor agonists. Physiologically based pharmacokinetic (PBPK) modeling predicts that delayed gastric transit prolongs time-to-maximum concentration (Tmax) and increases exposure to orally administered narrow-therapeutic-index medications [4]. Levothyroxine absorption is strongly influenced by gastric acidity, timing, and motility, and these effects may be magnified in post-thyroidectomy patients with no endogenous thyroid buffering. Preclinical work on GLP-1 receptor signaling also suggests central influences on hypothalamic function that may indirectly affect gastrointestinal motility patterns [6]. Additionally, bariatric surgery significantly alters levothyroxine pharmacokinetics, and delayed gastric emptying can further compound this variability, as documented in drug-interaction guidance from regulatory authorities [9]. Together, these mechanisms likely contributed to increased and inconsistent levothyroxine absorption in our patient.

Central modulation of the HPT axis

Preclinical studies demonstrate that GLP-1 receptors are expressed on TRH-producing neurons within the hypothalamic paraventricular nucleus, and that GLP-1 signaling can reduce TRH release at the median eminence, leading to downstream TSH suppression [6]. Clinical reviews also show that GLP-1 RAs may reduce TSH independently of weight loss [2], and early reductions in TSH have been reported within weeks of tirzepatide initiation [3].

In an athyreotic patient who depends entirely on exogenous thyroid hormone, even modest central suppression of TSH may unmask or intensify levothyroxine over-replacement, contributing to profound biochemical hyperthyroidism.

Additional thyroidal effects of tirzepatide

Beyond effects on levothyroxine metabolism, tirzepatide has been implicated in cases of drug-associated thyroiditis. A Cureus case report described a biphasic course of transient thyrotoxicosis followed by hypothyroidism after tirzepatide initiation [5]. This highlights that incretin-based therapies may produce a broad spectrum of thyroid perturbations, including metabolic, absorptive, central, and inflammatory effects.

Meta-analytic evidence supporting thyroid risk with GLP-1 RAs

A 2022 meta-analysis of randomized controlled trials found a 28% increased risk of overall thyroid disorders in GLP-1 RA users, although individual thyroid conditions remained rare [8].

This finding underscores the importance of enhanced monitoring in high-risk groups such as post-thyroidectomy patients on narrow-therapeutic-index thyroid hormone replacement.

## Conclusions

This case demonstrates how tirzepatide can significantly disrupt thyroid hormone stability in post-thyroidectomy patients maintained on fixed-dose levothyroxine, primarily through rapid weight loss, altered gastrointestinal absorption, and central effects on the hypothalamic-pituitary-thyroid axis. These combined mechanisms converted a previously stable levothyroxine dose into marked over-replacement, resulting in severe biochemical hyperthyroidism. Clinicians should closely monitor thyroid function every six to 12 weeks after initiating GLP-1/GIP receptor agonists in thyroid-dependent patients and anticipate the need for substantial levothyroxine dose reductions as weight changes occur. This case highlights the importance of recognizing incretin-based therapies as dynamic modifiers of thyroid hormone requirements, particularly in athyreotic patients, and underscores the need for further research to clarify best practices for levothyroxine management in individuals receiving GLP-1 receptor agonists.
